# Eu@C_72_: Computed Comparable Populations of Two Non-IPR Isomers

**DOI:** 10.3390/molecules22071053

**Published:** 2017-06-24

**Authors:** Zdeněk Slanina, Filip Uhlík, Shigeru Nagase, Takeshi Akasaka, Ludwik Adamowicz, Xing Lu

**Affiliations:** 1 State Key Laboratory of Materials Processing and Die & Mould Technology, School of Material Science and Engineering, Huazhong University of Science and Technology, Wuhan 430074, China; akasaka@tara.tsukuba.ac.jp (T.A.); lux@hust.edu.cn (X.L.); 2 Department of Physical and Macromolecular Chemistry, Faculty of Science, Charles University, Albertov 6, 128 43 Praha 2, Czech Republic; uhlik@sals.natur.cuni.cz; 3 Fukui Institute for Fundamental Chemistry, Kyoto University, Kyoto 606-8103, Japan; nagase@ims.ac.jp; 4 Department of Chemistry and Biochemistry, University of Arizona, Tucson, AZ 85721-0041, USA; ludwik@email.arizona.edu

**Keywords:** metallofullerenes, non-IPR fullerenes, relative populations of isomers

## Abstract

Relative concentrations of six isomeric Eu@C${}_{72}$—one based on the IPR C${}_{72}$ cage (i.e., obeying the isolated-pentagon rule, IPR), two cages with a pentagon–pentagon junction (symmetries $C_{2}$ and $C_{2v}$), a cage with one heptagon, a cage with two heptagons, and a cage with two pentagon–pentagon fusions—are DFT computed using the Gibbs energy in a broad temperature interval. It is shown that the two non-IPR isomers with one pentagon–pentagon junction prevail at any relevant temperature and exhibit comparable populations. The IPR-satisfying structure is disfavored by both energy and entropy.

## 1. Introduction

C${}_{72}$ has a special position among medium-size fullerenes [1,2,3] as it has not been isolated in the pristine form. A low solubility in conventional solvents or polymerization may be among the reasons for the difficulties [4]. As C${}_{72}$ could only be recorded [5] in the gas phase, its structure is not known. Still, C${}_{72}$ can act as a host cage for some metallofullerenes like Ca@C${}_{72}$ [6], La${}_{2}$@C${}_{72}$ [7], or La@C${}_{72}$ [8]. There is just one isolated-pentagon-rule (IPR) satisfying structure for C${}_{72}$, namely [1,2,3,9,10,11] with $D_{6d}$ symmetry and spiral-code number 11190. Nevertheless, it was pointed out [10] in conjunction with the Ca@C${}_{72}$ computations that two non-IPR (i.e., IPR-violating) structures with one pentagon–pentagon junction and the $C_{2}$ and $C_{2v}$ symmetries (code numbers [1,2,3,10,11] 10612 and 11188, respectively) produce Ca-endohedrals with lower energy than encapsulation in the IPR cage. It has been known for isomeric sets of fullerenes and metallofullerenes (e.g., Refs. [12,13,14,15,16]) that potential energy itself cannot generally decide stability order at high temperatures as the entropic part of the Gibbs energy becomes essential—this feature was also demonstrated for Ca@C${}_{72}$ [11] or La@C${}_{72}$ [8].

In this paper, density-functional theory (DFT) computations are carried out on another observed [17,18] C${}_{72}$-based metallofullerene, namely isomers of Eu@C${}_{72}$, in order to clarify their structures and stabilities and to enrich the knowledge on the whole C${}_{72}$-based endohedral family [19,20,21,22,23,24,25,26,27,28,29], considering IPR, classical non-IPR, and heptagon-containing structures [30].

## 2. Calculations

The computations started with DFT geometry optimizations, namely using Becke’s three parameter functional [31] combined with the non-local Lee–Yang–Parr correlation functional [32] (B3LYP). The basis set applied to C atoms is the standard 6-311G* basis [33] while Eu atom is treated in the SDD basis set [34] with the SDD effective core potential (the combined basis set is coded B3LYP/6-311G*∼SDD). The geometry optimizations were performed with the analytically constructed energy gradients. In the optimized B3LYP/6-311G*∼SDD geometries, the harmonic vibrational analysis was carried out with the analytical force-constant matrix. The inter-isomeric separation potential energies were further improved by the geometry optimizations at the B3LYP/6-311+G*∼SDD level. The electronic excitation energies were evaluated with the time-dependent (TD) DFT response-theory method [35,36]. The computations have been performed with the Gaussian 09 program package [37].

Relative concentrations (mole fractions) $x_{i}$ of *m* isomers can be expressed [38] through their partition functions $q_{i}$ and the enthalpies at the absolute zero temperature or ground-state energies $\Delta H_{0,i}^{o}$ (i.e., the relative potential energies corrected for the vibrational zero-point energies) by a compact formula:

(1) $$x_{i}=\frac{q_{i}exp\left[-\Delta H_{0,i}^{o}/\left(RT\right)\right]}{\sum_{j=1}^{m}q_{j}exp\left[-\Delta H_{0,j}^{o}/\left(RT\right)\right]},$$

where *R* is the gas constant and *T* the absolute temperature. Equation (1) is an exact formula that can be directly derived [38] from the standard Gibbs energies of the isomers, supposing the conditions of the inter-isomeric thermodynamic equilibrium. Rotational-vibrational partition functions were constructed from the calculated structural and vibrational data using the rigid rotator and harmonic oscillator (RRHO) approximation. No frequency scaling is applied as it is not significant [39] for the $x_{i}$ values at high temperatures. The geometrical symmetries of the optimized cages were determined by Gaussian 09 built-in procedure [37], and confirmed also by a procedure [40], which considers precision of the computed coordinates. The electronic partition function was evaluated by a direct summation. Finally, the chirality contribution [41] was included accordingly (for an enantiomeric pair its partition function $q_{i}$ is doubled).

However, the conventional RRHO treatment applied with Equation (1) is modified here by an approach for description of the encapsulate motions, namely in order to respect somehow [15,16] the findings that encapsulated atoms can exercise large amplitude motions, especially so at elevated temperatures (unless the motions are restricted by cage derivatizations). One can expect that if the encapsulate is relatively free, then, at sufficiently high temperatures, its behavior in different cages will bring about the same contribution to the partition functions. However, such uniform contributions would then cancel out in Equation (1). This simplification is called [15,16] free, fluctuating, or floating encapsulate model (FEM) and requires two steps. In addition to removal of the three lowest vibrational frequencies (belonging to the metal motions in the cage), the symmetries of the cages should be treated as the highest (topologically) possible. This step reflects the averaging effects of the large amplitude motions, showing themselves in the ${}^{13}$C-NMR spectra just by the observed high cage symmetries [15,16]. For example, for the Eu@C${}_{72}$ IPR isomer based on the $D_{6d}$ cage (Table 1), the $D_{6d}$ symmetry is indeed employed within the FEM scheme though its statical symmetry (i.e., after the geometry optimization) is only $C_{1}$ (Table 2). It has been established that the FEM treatment generally gives a better agreement [15,16] with the available observed data than the RRHO approach.

## 3. Results and Discussion

The four C${}_{72}$ cages originally selected [10] for Ca@C${}_{72}$ are also treated here: the IPR $D_{6d}$ cage (a), two non-IPR cages (b) and (c) with one pentagon–pentagon junction each with the symmetries $C_{2}$ and $C_{2v}$, respectively, and a $C_{s}$ structure with one heptagon (d). Selection of the structures is based on a common procedure that searches for low-energy dianionic cages (both Ca and Eu donate about two electrons to cage). Once such low-energy dianionic cages are selected, the next step is search for the lowest electrostatic-potential minimum, still in the charged cages, which suggests a starting position for the metal atom in following geometry optimizations. Moreover, other two interesting structures are added (Table 1) for the sake of illustration: a $C_{s}$ cage (e) with two heptagons pointed-out in Ref. [42], and a $D_{2}$ structure (code number 10611) with two pentagon–pentagon junctions (f) suggested in Ref. [25].

**Figure 1:**
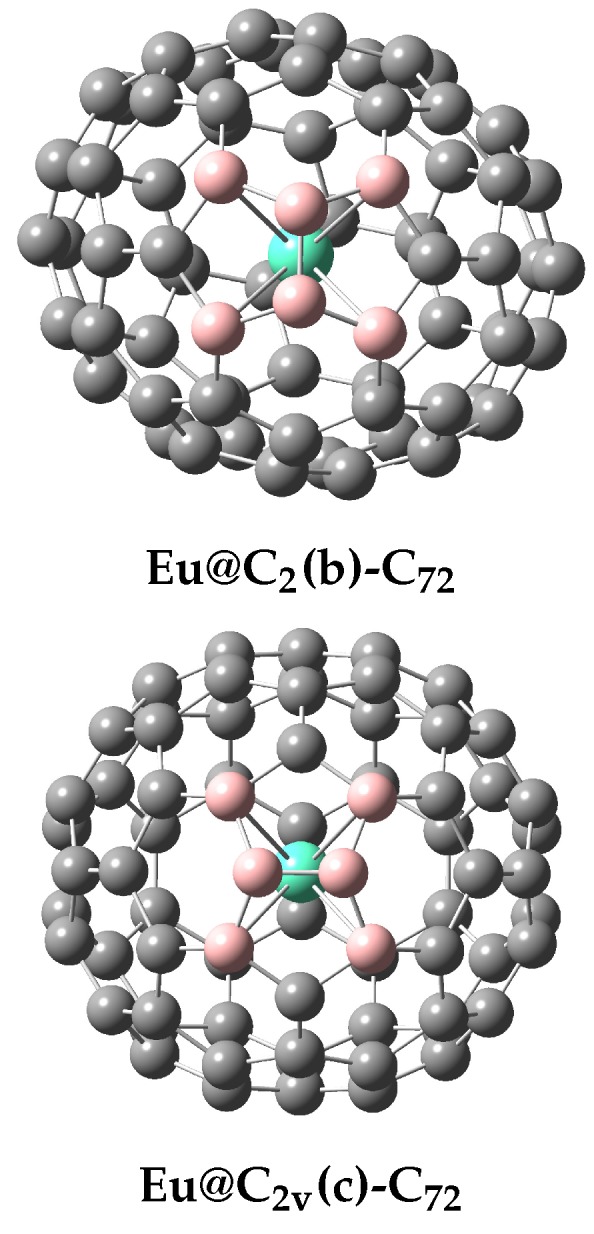
The B3LYP/6-311G*∼SDD optimized structures of (b) and (c) Eu@C${}_{72}$ (the carbons closest to Eu are in a lighter color and with the C-Eu links).

Table 1 reports the separation energetics computed for the B3LYP/6-311G*∼SDD and B3LYP/6-311+G*∼SDD optimized geometries. The energetics in both approaches are quite similar. The (c) structure based on the $C_{2v}$-symmetry cage with just one pentagon–pentagon fusion represents the potential-energy lowest isomer in both treatments, being closely followed by the (b) isomer employing the $C_{2}$ cage again with one 5/5 fusion. The remaining four cages are located at least more than 18 kcal/mol above the lowest one. The (d) isomer with one heptagon comes as the third potential-energy lowest species while other three endohedrals are at least 30 kcal/mol above the $C_{2v}$ stabilomer. Actually, the only IPR-cage based isomer is the highest-located in energy. The considered computational level is about the highest presently applicable and both the energies and structures should be quite reliable [43]. The all reported optimized structures are confirmed to be local energy minima by the vibrational analysis. The geometry optimizations produce some symmetry reductions (Table 2), which is rather common with endohedrals [44,45]. However, the symmetry of the two energy-lowest species does not undergo a reduction (Figure 1).

The computed shortest contacts between Eu and the cage carbons are rather uniform, spanning just a narrow interval between 2.643 and 2.712 Å (Table 2). The metal is never located in the cage center—it is placed relatively close to some portion of the cage. Formation of metallofullerenes is connected with a strong charge transfer from the metal to the cage [2,3]. The charge transfer is computed in Table 2 using the Mulliken atomic charges at the B3LYP/3-21G∼SDD level (this combination is known to give a good agreement with the observed charges [46] for metallofullerenes). The charge transfer is again rather uniform for all the isomers and is slightly larger than two electrons. The reported values agree with what previously found [47] for Eu@C${}_{74}$.

**Figure 2:**
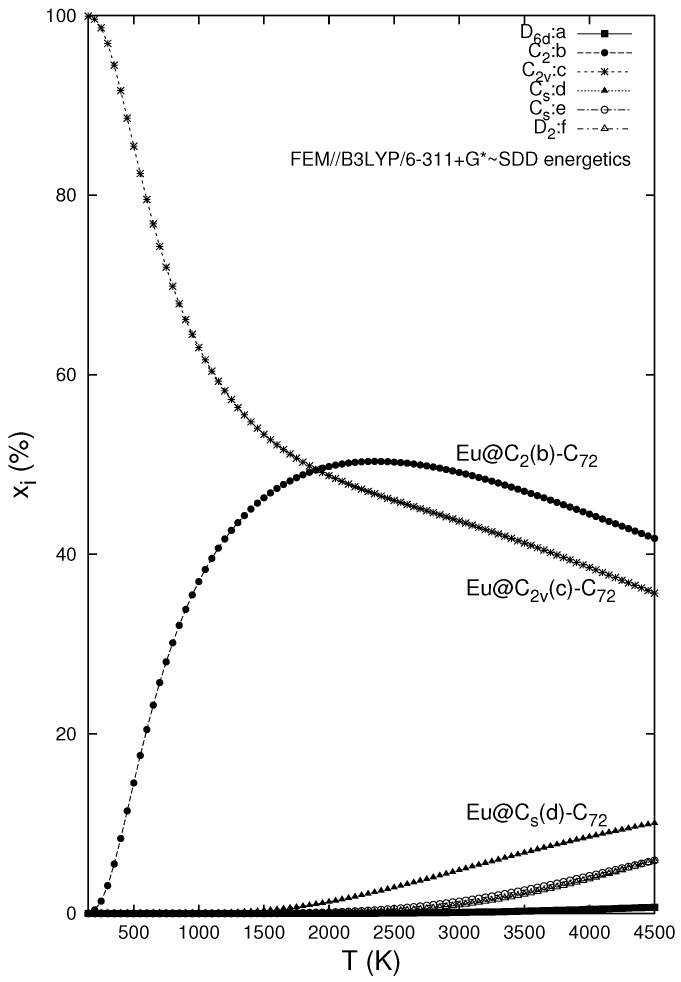
Relative concentrations of the Eu@C${}_{72}$ isomers (see Table 1) based on the B3LYP/6-311+G*∼SDD energetics, the B3LYP/6-311G*∼SDD entropy, and the FEM treatment.

Figure 2 presents the temperature development of the relative concentrations for the six Eu@C${}_{72}$ isomers evaluated within the FEM treatment in a wide temperature region. The enthalpy part of the Gibbs energy is based on the B3LYP/6-311+G*∼SDD energetics while the entropy contributions come only from the B3LYP/6-311G*∼SDD level (this is about the highest level that can presently be applied for the vibrational analysis of metallofulllerenes). It should be noted that the relative populations are derived in Equation (1) from a ratio that allows for a cancellation of possible systematic errors between the numerator and denominator. Although the temperature region where fullerene or metallofullerene electric-arc synthesis takes place is not well known, the recent observations [48] supply some arguments to expect it around or above 1500 K. Thus, the computed results should also be discussed in the temperature region. The lowest energy structure (c) based on the $C_{2v}$-symmetry cage is the most populated species only until a temperature of 1910 K. After this crossing point, the (b) structure with the $C_{2}$ cage becomes the most-populated one, however with a near-equimolarity of both endohedrals with one 5/5 fusion. All other structures are rather negligible, only the (d) endohedral with one heptagon, which is the third potential-energy lowest species, could represent a minor isomer. On the other hand, the only IPR-cage based structure (a) is the least populated. Its suppression should not be ascribed to the potential-energy term only. In fact, two other structures high in potential energy, (e) and (f), exhibit populations in a high-temperature limit approaching some 5%. Clearly enough, the IPR-cage based structure is suppressed not only by its energy but also by its unfavorable entropy term. One reason is its high symmetry, which in the FEM treatment also means a high rotational symmetry number (this factor would not operate in the simple RRHO approach that does not respect the averaging effects of the encapsulate large-amplitude motions).

Although rotational, vibrational, and electronic partition functions contribute to the overall picture, low vibrational frequencies are particularly important. Although we deal with the harmonic frequencies, Equation (1), owing to its already mentioned convenient form, allows for at least partial cancellation of anharmonicity corrections. This expectation is supported by model computations [49] for C${}_{6}$ (and also by a good agreement with the observed concentration ratios available for several fullerenic systems [15,16,50]). The enthalpy–entropy interplay can be visualized by the following data. If all the partition functions are neglected together with the zero-point vibrations, Equation (1) is reduced to the simple Boltzmann isomeric factors governed purely by the potential energy. At a selected temperature of 2000 K, Equation (1) gives the concentration ratios (a):(b):(c):(d):(e):(f) = 0.005:49.8:48.8:1.3:0.1:0.04 (Figure 2). If the entropy contributions are neglected and only the Boltzmann factors with the B3LYP/6-311+G*∼SDD potential-energy terms are applied, the concentration ratios are changed considerably (a):(b):(c):(d):(e):(f) = 0.007:35.1:64.3:0.6:0.03:0.008. Interestingly, the simple Boltzmann factors can never cross in any isomeric system, and, thus, they are not convenient for the relative-population evaluations.

The computations predict co-existence of two major isomers for Eu@C${}_{72}$, both having non-IPR cages with one pentagon–pentagon junction each. A third, minor isomer has a cage with one heptagon (i.e., a non-classical fullerene cage [13,51]), while the IPR-cage based Eu-endohedral is ruled out. The prediction agrees with Bucher et al.’s observations [14,15] of two Eu@C${}_{72}$ isomers (though yet without structural conclusions based on NMR or X-ray crystal analysis). The present computations, however, refer to the equilibrium gas-phase conditions and do not cover such aspects like polymerization, solubility or reactions with solvent. The computations also do not deal with the relative stabilities compared to other Eu-metallofullerenes as the question involves pressure considerations [52,53,54]. However, another issue is a possibility of kinetic control of the formation process, including a catalytic enhancement [55,56]. The study nevertheless expands the family of isomeric metallofullerene systems [57,58,59,60] for which the Gibbs-energy treatment produces an encouraging agreement with available observations, and, thus, other C${}_{72}$-based endohedrals [17,18,61,62] should in future be treated with the present or even higher approaches too.

## Figures and Tables

**Table 1:** Eu@C${}_{72}$ relative potential energies $\Delta E_{pot,rel}$ [kcal/mol].

Species ${}^{a}$	Point	ΔE_*pot,rel*_	B3LYP/6-311+G*∼SDD
	Group ${}^{b}$	B3LYP/6-311G*∼SDD	
(a) IPR	$D_{6d}$	36.41	36.04
(b) 5/5 fusion	$C_{2}$	2.35	2.40
(c) 5/5 fusion	$C_{2v}$	0.0	0.0
(d) 7-ring	$C_{s}$	18.59	18.56
(e) two 7-rings	$C_{s}$	29.99	29.94
(f) two 5/5 fusions	$D_{2}$	35.80	35.73

${}^{a}$ For (b), (c)—see Figure 1. ${}^{b}$ Symmetry of the empty cage.

**Table 2:** The shortest Eu-C contact d${}_{Eu-C}$ and metal charge $q_{Eu}$ for the Eu@C${}_{72}$ isomers.

Species ${}^{a}$	Symmetry ${}^{b}$	d${}_{\mathrm{Eu}-C}$ [Å] ${}^{c}$	$q_{\mathrm{Eu}}$ ${}^{d}$
(a) IPR	$C_{1}$	2.705	2.207
(b) 5/5 fusion	$C_{2}$	2.712	2.277
(c) 5/5 fusion	$C_{2v}$	2.643	2.201
(d) 7-ring	$C_{1}$	2.648	2.277
(e) two 7-rings	$C_{s}$	2.567	2.206
(f) two 5/5 fusions	$C_{2}$	2.690	2.270

${}^{a}$ For (b), (c)—see Figure 1. ${}^{b}$ Symmetry of the fully optimized ${}^{c}$ endohedral. ${}^{c}$ Computed in the B3LYP/6-311G*∼SDD optimized structures. ${}^{d}$ The B3LYP/3-21G∼SDD Mulliken atomic charge ${}^{c}$ (given as multiply of the elementary charge).
